# Effect of the clinical decision assessment system on clinical outcomes of delirium in hospitalized older adults: study protocol for a pair-matched, parallel, cluster randomized controlled superiority trial

**DOI:** 10.1186/s13063-023-07607-3

**Published:** 2023-09-11

**Authors:** Jiamin Wang, Sen Niu, Ying Wu

**Affiliations:** 1 School of Nursing, Beijing University of Chinese Medicine, Beijing, 102488 China; 2 School of Nursing, Capital Medical University, 10 You-an-Men Wai Xi-Tou-Tiao, Fengtai District, Beijing, 100069 China

**Keywords:** Delirium, Older adults, Clinical outcome, Clinical decision assessment system, Protocol

## Abstract

**Background:**

Prompt recognition of delirium is the first key step in its proper management. A previous study has demonstrated that nurses’ delirium screening using the usual paper version assessment tool has no effect on clinical outcomes. Clinical decision assessment systems have been demonstrated to improve patients’ adherence and clinical outcomes. Therefore, We developed a clinical decision assessment system (3D-DST) based on the usual paper version (3-min diagnostic interview for CAM-defined delirium), which was developed for assessing delirium in older adults with high usability and accuracy. However, no high quality evidence exists on the effectiveness of a 3D-DST in improving outcomes of older adults compared to the usual paper version.

**Methods:**

A pair-matched, open-label, parallel, cluster randomized controlled superiority trial following the SPIRIT checklist. Older patients aged 65 years or older admitted to four medical wards of a geriatric hospital will be invited to participate in the study. Prior to the study, delirium prevention and treatment interventions will be delivered to nurses in both the intervention and control groups. The nurses in the intervention group will perform routine delirium assessments on the included older patients with 3D-DST, while the nurses in the control group will perform daily delirium assessments with the usual paper version. Enrolled patients will be assessed twice daily for delirium by a nurse researcher using 3D-DST. The primary outcome is delirium duration. The secondary outcomes are delirium severity, incidence of delirium, length of stay, in-hospital mortality, adherence to delirium assessment, prevention, and treatment of medical staff.

**Discussion:**

This study will incorporate the 3D-DST into clinical practice for delirium assessment. If our study will demonstrate that 3D-DST will improve adherence with delirium assessment and clinical outcomes in older patients, it will provide important evidence for the management of delirium in the future.

**Trial registration:**

Chinese Clinical Trial Registry, Identifier: ChiCTR1900028402. https://www.chictr.org.cn/showproj.aspx?proj=47127. Protocol version: 1, 29/7/22.

**Supplementary Information:**

The online version contains supplementary material available at 10.1186/s13063-023-07607-3.

## What is already known

- Delirium screening using the usual paper version assessment tool by nurses has no effect on clinical outcomes.
- Clinical decision assessment systems have been demonstrated to improve patients’ adherence and clinical outcomes.
- No high-quality evidence exists on the effectiveness of a 3D-DST in improving outcomes of older adults compared to the usual paper version.

## What this paper adds

- A cluster randomized controlled trial will be conducted to evaluate the effect of the 3D-DST on clinical outcomes.
- Findings will improve policy and practice for delirium management.

## Introduction

Delirium is a common neurocognitive syndrome in hospitalized older adults with fluctuating symptoms [1, 2]. The incidence of delirium in hospitalized older adults has been reported to be 10% ~ 40% in different settings [3–6]. Delirium during hospitalization will increase the risk of falls, decrease cognitive function, aggravate frailty, and increase mortality in older adults [1, 7]. The severity of adverse effects was positively correlated with the severity and duration of delirium [8]. Prompt recognition of delirium is the first key step in its proper management.

Relevant guidelines for the routine assessment and management of delirium have been issued [9, 10], and several studies have found that delirium assessment combined with multi-component intervention can effectively improve clinical outcomes of patients [11, 12]. However, the majority of patients are not routinely monitored for delirium, guidelines are poorly implemented and hindering timely prevention and management [13]. The possible reasons for the low adherence to delirium assessment are multiple but mainly due to the implementation barriers, such as the monitoring instrument is not routinely used, the complicated procedure of the evaluation instrument, the long evaluation time required for delirium increases the workload of the nurse, etc. [14–16]. A prospective study also demonstrates that routine delirium detection by bedside nurses using the usual paper version assessment tool does not improve clinical outcomes [17].

Clinical decision assessment systems have been widely used in the medical field and have been demonstrated to improve patients’ adherence and clinical outcomes [18, 19]. We have developed a clinical decision assessment system (3D-DST) based on the 3-min diagnostic interview for CAM-defined delirium (3D-CAM), which is developed for the assessment of delirium in older adults with high sensitivity and specificity [20]. 3D-DST simplified the evaluation process of the 3D-CAM paper version, the usability of 3D-DST is significantly better than that of the paper version when used by bedside nurses, and the assessment time has been shortened by 2.1 min [20]. However, no high quality evidence exists on the effectiveness of 3D-DST in improving delirium identification and clinical outcomes of older adults compared to the usual paper version.

Therefore, we hypothesize that the use of the 3D-DST will improve the adherence to delirium assessment and management by clinical staff, and reduce the duration and the severity of delirium.

## Objectives

The primary objective is to determine the effect of routine delirium assessment using the 3D-DST by bedside nurses on the duration of delirium.

The secondary objectives are to determine the impact of routine delirium assessment using the 3D-DST by bedside nurses on delirium severity, incidence, length of stay, in-hospital mortality, adherence to delirium assessment, prevention, and treatment of medical staff.

## Methods

### Reporting guideline

**Fig. 1:**
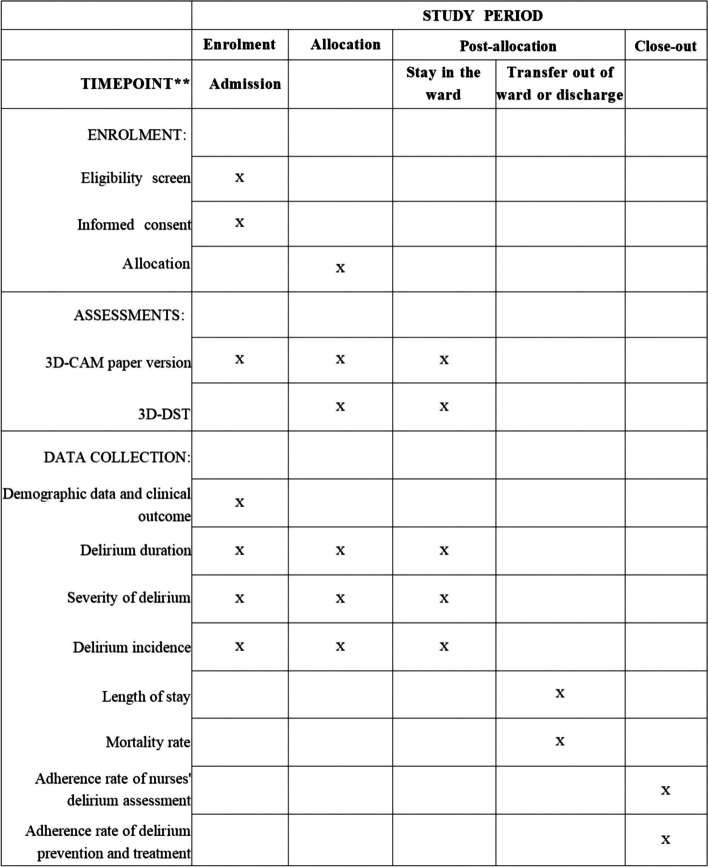
Schedule for enrolment, data collection, assessments, interventions and outcome measures

Following the defining standard protocol items for clinical trials (SPIRIT 2013) [21]. SPIRIT Checklist is included as Additional file 1 and the SPIRIT Figure is shown in Fig. 1.

### Design and setting

This study is a pair-matched, open-label, parallel, cluster randomized controlled superiority trial with a 1:1 allocation ratio. Cluster randomization minimizes group contamination and accounts for intraclass correlation [22]. Four medical wards (two respiratory and two neurology wards) of a geriatric hospital in China will be invited to participate in the study. The neurology and respiratory wards receive approximately 20 and 40 older adults per month, respectively. This study was registered on the Chinese Clinical Trial Registry (registration number: ChiCTR1900028402).

### Participants

The participants are older patients continuously admitted to four general medical wards. Patients should be: 1) age ≥ 65 years old, 2) able to communicate effectively in Mandarin, 3) stay in the hospital longer than two days. Exclusion criteria are: 1) severe hearing or visual impairment, 2) refuse to participate in this study, 3) continuous coma during the study period, 4) severe dementia which is defined as a diagnosis documented in the medical record because such patients cannot be assessed for delirium. This study was approved by the ethics committee of the Capital Medical University and the study hospital. Patients who meet the inclusion criteria will be informed about the study protocol, and written informed consent will be obtained from the older participant or their legal representatives by the first author. Three supervisors (member of the Ethics Committee, administrators of the research program and education department in the hospital) will monitor the accuracy and feasibility of the research process once a year.

### Instruments for delirium assessment

#### 3D-CAM paper version

A total of 22 items and four features are included in the 3D-CAM assessment. Four features are acute change and fluctuation of mental status (feature one), inattention (feature two), disorganized thinking (feature three), and altered level of consciousness (feature four) [23]. Delirium is positive if feature one and feature two are present with either feature three or feature four. The 3D-CAM has been translated into Chinese [24]. Previous evidence has shown highly acceptable sensitivity and specificity of 3D-CAM for routine delirium assessment when used by nurse researchers [24].

#### 3D-DST

**Fig. 2:**
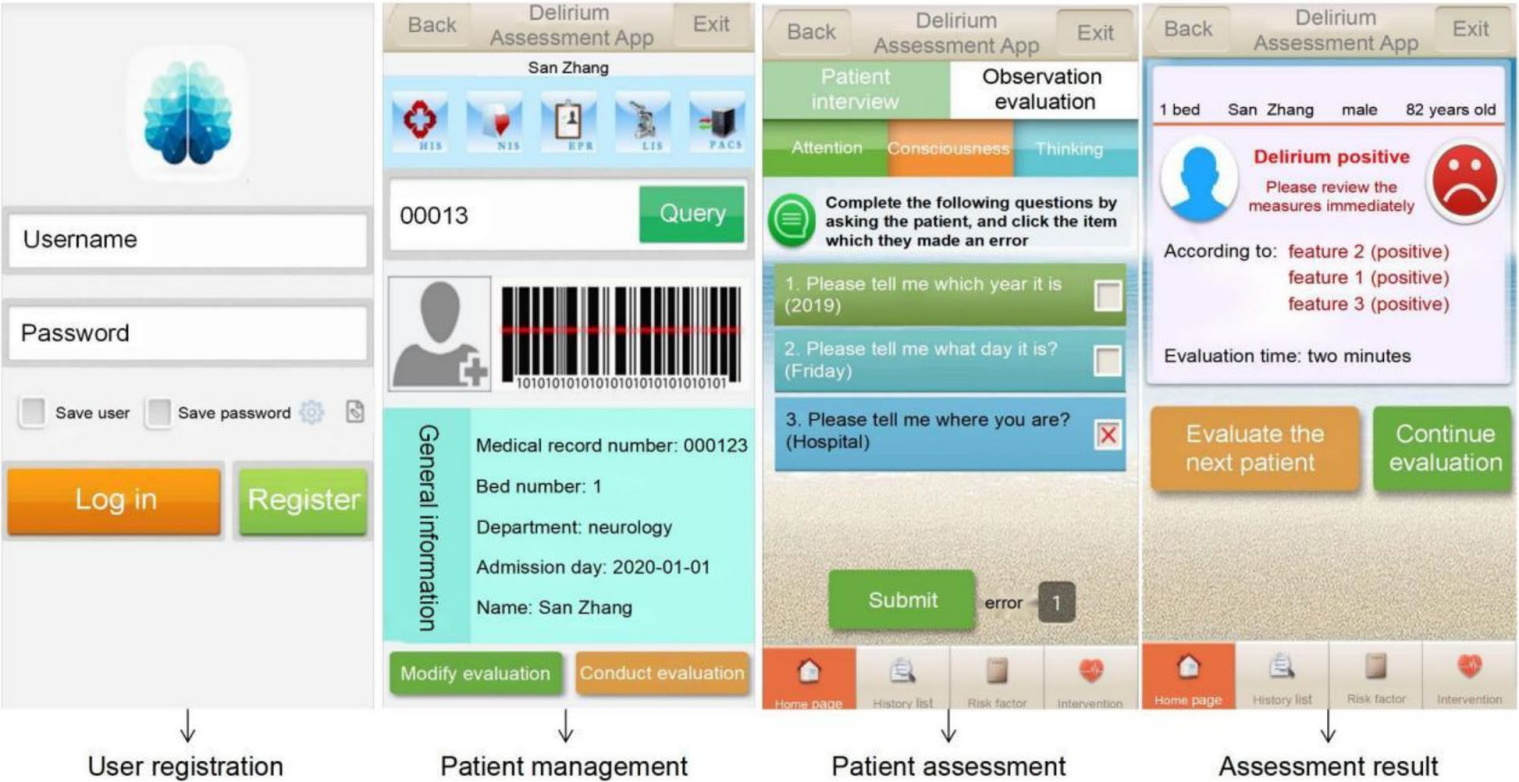
Interfaces of the 3D-DST

Based on the paper version, the 3D-DST was programed to fit on Android-compatible smartphones through three steps include analysis of problems in the use of the 3D-CAM, design of the 3D-DST (evaluation process analysis and optimization, and user interface design), architecture and development of 3D-DST [25]. A multidisciplinary team that included experts with rich experience in delirium assessment, bedside nurses, nurse researchers, software engineers and user interface designers were formulated to develop the 3D-DST. Three assessment modules (inquiry assessment, observation assessment and selective assessment), nine assessment interfaces with built-in reminders to guide nurses to complete the assessment, and 16 result interfaces were included in the final 3D-DST version (Fig. 2) [20]. The 3D-DST final version has fully addressed the problems that were identified when using the 3D-CAM by bedside nurses, including human error, incorrect understanding of the item content, and incomplete nursing records when the 3D-CAM was used by nurses. In the usability testing, a total of 432 delirium assessments on 148 older adults were performed by 72 bedside nurses [25]. Our previous research had shown that the 3D-DST had good usability when used by bedside nurses, reduced human error, and saved evaluation time compared to the paper version (2.3 min vs. 4.4 min) [25].

#### CAM-S

Inouye et al. have developed a delirium severity scoring system (CAM-S) [26]. CAM-S can only be used to assess the severity of delirium and not be used to diagnose delirium. The simple version of CAM-S includes the following items: acute episodes or symptom fluctuations, impaired attention, incoherent thinking, and changes in consciousness. The results show that the overall agreement of the CAM-S scale is 97%. The score range is 0 to 7 ( 0 = normal, 1 = mild delirium, 2 = moderate delirium, and 3–7 = severe delirium [26]. The CAM-S has been translated into Chinese with high reliability and validity and can be used as an effective tool to evaluate the severity of delirium [27].

### Cluster randomization and blinding

Before randomization, the wards that participated in the study will be pair matched according to the specialty. Within each of the two pairs, wards will be randomly assigned (1:1) to the intervention or control group using random numbers generated by the computer, which indicates that each group will contain one neurology and one respiratory ward. The medical staff who will be involved in the study only know the protocol of their ward, but not be aware of the protocol of other wards and the hypothesis of the study. Due to the allocation sequence generating, participants’ enrollment, participants’ assignment, data and outcome collection will be done by a nurse researcher, therefore the protocol will not be blinded to the nurse researcher. However, the nurse researcher and the bedside nurse will be unaware of each other's assessment results of delirium.

### Sample size

The primary outcome is delirium duration. We expect that the delirium duration of the intervention group will be one day shorter than that of the control group, with a standard deviation of 1.8 [28], α of 0.05, and intraclass correlation coefficient of 0.00001. Our study will be estimated to provide more than 80% power, at least five delirium-positive patients in each group (ward), and 20 delirium-negative patients will be needed. According to our previous study, the incidence of delirium is approximately 10%, with an estimated 10% of patients dropping out during the study, at least 202 older patients will be needed. Patients will be given a detailed explanation of the benefits they will receive from participating in the study to reach the target sample size.

### Intervention and implementation

**Fig. 3:**
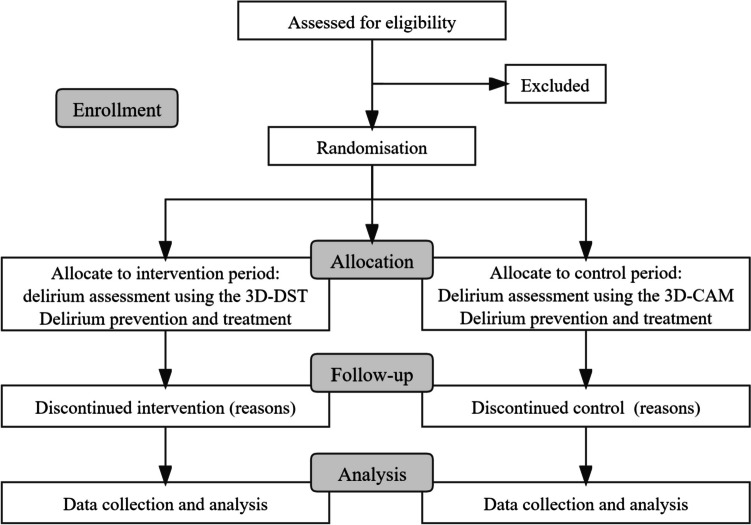
CONSORT flow diagram

The intervention includes assessment, prevention, and treatment of delirium implemented by nurses and clinicians (Fig. 3).

#### Delirium assessment

Prior to the study, bedside nurses of the control and intervention groups will be trained respectively. The nurses in the intervention group will perform routine delirium assessments on the included older patients with 3D-DST, while the nurses in the control group will perform daily delirium assessments with the 3D-CAM paper version. The training will include the concept of delirium, clinical manifestations, the training manual of the 3D-DST or 3D-CAM, which will last for 30 min. Due to the characteristic of fluctuating of delirium, enrolled patients will be evaluated for delirium twice a day by bedside nurses during the forenoon and night.

#### Delirium prevention and treatment

Prior to the study, our team summarized prevention and treatment interventions according to the NICE (National Institute for Health and Clinical Excellence) guidance and expert consensus in older patients with postoperative delirium by Chinese and American experts [9, 10, 29]. Based on the clinical situation and discussion with geriatric experts in the participating hospital, the final version of the delirium prevention and treatment interventions are determined. The researcher delivers the paper version of interventions to the head nurses in both the intervention and control groups. The intervention details are as follows (Table 1):

A. Prevention: monitoring and managing of hypoxemia, early mobility and/or physical rehabilitation, early mobility and/or physical rehabilitation, pain assessment, and management.
B. Treatment: identifing and managing the possible underlying cause, pharmacologic treatments, information and assessment, and interventions for people in whom delirium does not resolve.

**Table 1:** Interventions for delirium prevention and treatment

Intervention	Details
Monitoring and management of hypoxemia	Assess for hypoxia and optimize oxygen saturation if necessary
Early mobility and/or physical rehabilitation	Encourage the person to mobilize soon after surgery, walk (provide walking aids if needed-these should be accessible at all times), encourage the person to carry out and active range of motion exercises, even if unable to walk, the intervention of the rehabilitation department
Medication reductions	Reduce the types of drugs you use, avoid medications that induce delirium, use alternative therapies or consult a psychiatrist to adjust dosage (anticholinergic drug, anticonvulsant drug, tricyclic antidepressant, antihistamine drug, antiparkinson drug, antipsychotic drug, benzodiazepines, H2 receptor antagonists, non-benzodiazepine, opioid analgesics)
Pain assessment and management	Optimize postoperative pain control, starting and reviewing appropriate pain management in any person in whom pain is identified or suspected. Preferably with nonopioid pain medications to minimize pain in older adults to prevent delirium
In people diagnosed with delirium, a medical evaluation should be performed to identify and manage underlying contributors to delirium	
Pharmacologic treatments	The prescribing practitioner may use antipsychotics at the lowest effective dose for the shortest possible duration to treat patients who are severely agitated or distressed, and are threatening substantial harm to self and/or others A. Hyperactive delirium: the use of antipsychotics (e.g., haloperidol, risperidone, olanzapine, quetiapine, or ziprasidone) at the lowest effective dose for the shortest possible duration may be considered to treat delirious patients who are severely agitated or distressed or who are threatening substantial harm to self and/or others. Cholinesterase inhibitors should not be newly prescribed to prevent or treat postoperative delirium. Benzodiazepines should not be used as the first-line treatment of agitation associated with delirium. The principles of drug treatment are as follows: a. Monotherapy is better than combination drug therapy b. Start at the lowest clinically appropriate dose and titrate cautiously according to symptoms c. Select drugs with low anticholinergic activity d. Stop medication immediately e. Continued application of nonpharmacologic intervention, identify and manage the possible underlying cause(s) B. Hypoactive delirium: antipsychotics and benzodiazepines should be avoided for treatment of hypoactive delirium C. Mixed delirium: when hyperactive delirium occurs, treated according to the treatment method of hyperactive delirium. When hypoactive delirium occurs, treated according to the treatment method of hypoactive delirium
Information and assessment	Offer information to people who are at risk of delirium or who have delirium, and their family and/or carers. Inform them that delirium is common and usually temporary. Describe people’s experience of delirium. Encourages people at risk and their families and/or carers to tell their healthcare team about any sudden changes or fluctuations in behavior. Encourage the person who has had delirium to share their experience of delirium with the healthcare professional during recovery
For people in whom delirium does not resolve	Re-evaluate for underlying causes. Follow up and assess for possible dementia

### Adherence/termination

As the adherence of medical staff in implementing the interventions is one of the outcomes of this study, the researchers will not intervene in the adherence of medical staff. If participants choose to withdraw, transfer out of the ward, die, or discharge, they will stop receiving the interventions. The reason will be recorded if the patient fails to complete the evaluation during the evaluation period.

### Study outcomes

Delirium positive is defined as the occurrence of delirium assessed by a nurse researcher using the 3D-DST. Enrolled patients will be assessed twice daily for delirium by a nurse researcher using the 3D-DST for the same period of time as the nurses in the wards. Delirium duration, delirium severity, incidence of delirium, length of stay, in-hospital mortality, delirium assessment, delirium prevention, and treatment interventions performed by medical staff will be recorded by the nurse researcher.

The primary outcome of the study is delirium duration: defined as the number of delirium positive days in older adults between admission and death/transfer from the ward.

Secondary outcomes include six aspects.

1. The severity of delirium: defined as the score of the CAM-S scale evaluated by the nurse researcher.
2. Delirium incidence: defined as the percentage of delirium-positive patients accounting for the total number of included patients from admission to death/transfer out of the ward who were assessed by the nurse researcher using the 3D-DST.
3. Length of stay: defined as the total number of of hospital days from admission to the day of death/transfer.
4. Mortality rate: defined as the percentage of patients who died during hospitalization account for the total number of enrolled patients.
5. The rate of adherence to delirium assessment: defined as the number of delirium assessments by the nurse as a percentage of the number of theoretically required assessments between patient’s admission and death/transfer out of the ward.
6. The rate of adherence to delirium prevention and treatment: defined as the number of preventive and treatment interventions implemented by nurses and clinicians as a percentage of the number of theoretically required interventions between patient’s admission and death/transfer from the ward.

### Data collection

A nurse researcher enrolls the older patients according to the inclusion and exclusion criteria and collect demographic data at admission, including sex, age, ethnicity, education level, marital status, smoking, and drinking. Clinical data includes diagnosis of major diseases, visual acuity level, hearing level, cognitive function, physical function, depression, and comorbidity. The mini-mental state examination (MMSE) is used to assess cognitive function, the total score is 0–30, and a higher score indicates better cognitive function [30]. The Geriatric depression scale (GDS-15) is used to assess depression, the overall score is 0–15, with less than 8 indicating depression [31]. The Charlson comorbidity index (CCI) is used to assess the comorbidity index, and a higher score indicates more underlying diseases in older people [32]. The Modified barthel index (MBI) is used to assess the activities of daily living at admission by the nurse researcher, and a higher score indicates better mobility. Restraint use during hospitalization will also be recorded [33].

The delirium assessment data will be stored in a smartphone with 3D-DST installed, and the data can be exported. Patients' general and clinical data is collected using questionnaires, and the data will be input into SPSS by two researchers and checked for errors. The data is stored by the investigator and is confidential before, during and after the trial. Only the investigators responsible for the outcome collection and supervisors of this study have access to the data according to the protocol.

Prior to the study, a study group is established, all adverse events will be reviewed regularly by the researchers, and a meeting of the researchers will be convened if necessary to assess the risks and benefits of the study. All adverse events will be recorded in detail, properly handled, and followed up until the adverse events are properly resolved. Serious adverse events will be reported to the ethics committee and authorities according to regulations.

### Statistical analysis

SPSS 22.0 (SPSS Inc., Chicago, Illinois, USA) will be used for statistical analysis. Multiple imputation will be used to fill in missing data using the R software. A *P*-value of 0.05 will be defined as statistically significant. The continuous data will be tested for normality and homogeneity of variance by the "One-Sample Kolmogorov Smirnov Test" and the "Homogeneity of Variance Test". Mean and standard deviation (SD) will be used for normally distributed continuous variables, median and interquartile range (IQR) will be used for continuous variables with non-normal distribution. The rank sum test and *t*-test will be used to compare the demographics and clinical characteristics of different groups. Categorical variables will be expressed in frequency and percentage, and the Chi-square test or Fisher's Exact Test will be used for comparing variables between different groups. The effects on clinical outcomes will be analyzed by a generalized linear mixed model. Subgroup analysis will be performed to eliminate the effect of confounding factors (age, gender, education level, comorbidity, marital status, smoking, drinking, cognitive function, physical function, depression, and comorbidity). Data will be analyzed according to the per-protocol principle. The preliminary analysis will be conducted after the start of the study (three months, one year, close-out).

## Discussion

Routine assessment of delirium is essential for early identification of delirium, including identifying positive patients and their risk factors for delirium, thus improving clinical outcomes. However, the effectiveness of routine delirium assessment using a clinical decision assessment system on clinical outcomes by bedside nurses has not been rigorously evaluated. In this study, a smartphone-based clinical decision assessment system (3D-DST) will be incorporated into clinical practice. The effect of the use of 3D-DST on the adherence of nurses' delirium assessment, adherence to delirium prevention and treatment measures as well as the clinical outcomes of older patients will be evaluated through a cluster randomized controlled trial. If our study demonstrates that the use of 3D-DST will improve the adherence to delirium assessment by nurses, and improve clinical outcomes of older patients, it may significantly improve the care and management of delirium patients in hospital.

The design is a parallel, cluster randomized controlled trial, which is of higher quality than the results of non-randomized trials and can provide strong evidence for the effectiveness of the implementation protocol. In addition, participating wards will be paired on the specialty, effectively reducing the risk of contamination and bias between study groups.

In recent years, there has been increasing concern among healthcare professionals about delirium, and many guidelines and associated professional organizations have also recommended routine assessment of delirium (preferably each shift) [14]. However, the adherence to delirium assessment with evaluation tools of nurses is still poor, and delirium assessment has not been incorporated into clinical practice [34]. Therefore, improving adherence to nurses' routine delirium assessment is critical to change the clinical outcomes of patients. Although 3D-CAM has been recognized as a highly acceptable assessment tool for diagnosing delirium in older adults, our previous study has demonstrated that human errors and evaluation problems may occur when the 3D-CAM is used by nurses [35]. Therefore, we developed the 3D-DST based on the 3D-CAM, and the results showed that the usability of 3D-DST was significantly higher than that of 3D-CAM. Therefore, this study will be conducted to compare the adherence to delirium assessment using the 3D-DST and 3D-CAM when used by nurses and evaluate its impact on clinical outcomes.

Improving clinical outcomes in older patients should be combined with preventive and treatment measures and routine assessments. The preventive and treatment measures that will be adopted in this project were summarized based on guidelines and expert recommendations on delirium. The feasible interventions, including pharmacological and non-pharmacological interventions, have been determined through complete discussion with geriatricians and managers of the study hospital. Therefore, the intervention program will be feasible.

Limitations of this study. This study will require the nurse researcher to collect outcomes in all the participating wards, it will not be possible for the nurse researcher to keep blinded to the protocol implemented by nurses, so an open-label trial is designed. However, clinical staff will keep blinded to the results performed by the nurse researcher, an open-label design will not affect the results. Although our study will be conducted in only one hospital, the results may have limited generalization. However, the main conclusions of this study will provide a critical preliminary research basis for the management of delirium in the future.

### Trial status

Enrollment is ongoing. Recruitment has began in September 2020 and will be expected to conclude in November 2023. The target enrollment for the study is 202 participants.

### Supplementary Information

**Additional file 1.**

## Data Availability

The datasets analyzed during the current study and statistical code are available from the corresponding author on reasonable request, as is the full protocol.

## Abbreviations

3D-DST: Clinical decision assessment system based on the 3-min diagnostic interview for CAM-defined delirium.
3D-CAM: 3-Minute diagnostic interview for CAM-defined delirium.
CAM-S: Delirium severity scoring system.
NICE: National Institute for Health and Clinical Excellence
